# Ethyl 4-[(3,5-di-*tert*-butyl-2-hy­droxy­benzyl­idene)­amino]­benzoate

**DOI:** 10.1107/S1600536810041383

**Published:** 2010-10-23

**Authors:** Raied Mustafa Shakir, Azhar Ariffin, Seik Weng Ng

**Affiliations:** a Department of Chemistry, University of Malaya, 50603 Kuala Lumpur, Malaysia

## Abstract

The title compound, a Schiff base, C_24_H_31_NO_3_, has a substituted aromatic ring at both ends of the azomethine linkage and these make a dihedral angle of 24.9 (1)°. There is an intra­molecular hydrogen bond between the hy­droxy group (donor) and the N atom of themazomethine linkage.

## Related literature

For the use of the methyl ester analog of the title compound as a second-harmonic generation material, see: Sliwa *et al.* (2008 ▶).
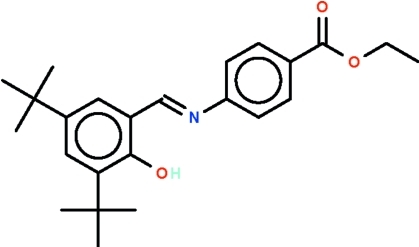


## Experimental

### 

#### Crystal data

C_24_H_31_NO_3_
*M* *_r_* = 381.50
Monoclinic, 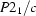

*a* = 18.4789 (18) Å
*b* = 10.7194 (11) Å
*c* = 10.7768 (10) Å
β = 97.437 (2)°
*V* = 2116.7 (4) Å^3^
*Z* = 4
Mo *K*α radiation
μ = 0.08 mm^−1^
*T* = 100 K
0.30 × 0.05 × 0.05 mm

#### Data collection

Bruker SMART APEX diffractometer
19941 measured reflections
4855 independent reflections
3123 reflections with *I* > 2σ(*I*)
*R*_int_ = 0.065

#### Refinement

*R*[*F* ^2^ > 2σ(*F* ^2^)] = 0.053
*wR*(*F* ^2^) = 0.142
*S* = 1.01
4855 reflections
257 parameters
1 restraint
H atoms treated by a mixture of independent and constrained refinement
Δρ_max_ = 0.64 e Å^−3^
Δρ_min_ = −0.26 e Å^−3^

### 

Data collection: *APEX2* (Bruker, 2009 ▶); cell refinement: *SAINT* (Bruker, 2009 ▶); data reduction: *SAINT*; program(s) used to solve structure: *SHELXS97* (Sheldrick, 2008 ▶); program(s) used to refine structure: *SHELXL97* (Sheldrick, 2008 ▶); molecular graphics: *X-SEED* (Barbour, 2001 ▶); software used to prepare material for publication: *publCIF* (Westrip, 2010 ▶).

## Supplementary Material

Crystal structure: contains datablocks global, I. DOI: 10.1107/S1600536810041383/fl2320sup1.cif

Structure factors: contains datablocks I. DOI: 10.1107/S1600536810041383/fl2320Isup2.hkl

Additional supplementary materials:  crystallographic information; 3D view; checkCIF report

## Figures and Tables

**Table 1:** Hydrogen-bond geometry (Å, °)

*D*—H⋯*A*	*D*—H	H⋯*A*	*D*⋯*A*	*D*—H⋯*A*
O1—H1⋯N1	0.87 (1)	1.80 (2)	2.609 (2)	154 (3)

**Fig. 1.**
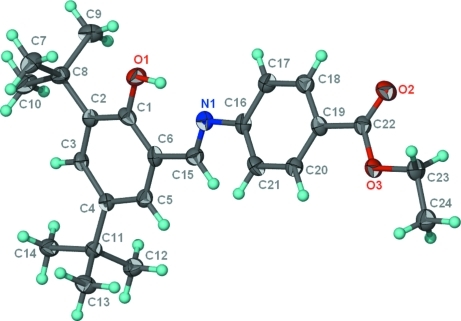
Thermal ellipsoid plot (Barbour, 2001) of the C24H31NO3 at the 70% probability level; hydrogen atoms are drawn as spheres of arbitrary radius.

**Fig. 2.**
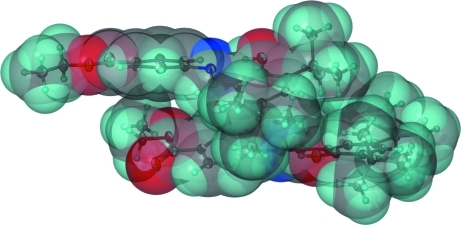
Van der Waals packing of two adjacent molecules.

## supplementary crystallographic information

### Comment

The Schiff base, methyl 4-(di-3,5-*tert*-butyl-2-hydroxybenzylideneamino)benzoate, is a material suitable for second-harmonic generation as it has electron-donating and electron-withdrawing components that are critical for the manifestation of a permanent dipole (Sliwa *et al.*, 2008). Replacing the methyl group with an ethyl moiety leads to (I), an intensely orange-colored compound (Scheme I, Fig. 1) that crystallizes in a centric space group and is, therefore, not suitable as an SHG material. The azomethine bond has an *E*-configuration; the two aromatic rings are aligned at 24.9 (1) °. The compound is neutral as the hydroxy group bears a hydrogen atom which is a donor in an intra-molecular H bond to the azomethine nitrogen atom (Table 1). There are no important intermolecular contacts; on the other hand, the compound appears to pack in such a way as to accomodate the bulky *t*-butyl groups as far as possible (Fig. 2).

### Experimental

Ethyl 4-aminobenzoate (0.35 g) dissolved in ethanol (5 ml) was added to 3,5-di-*tert*-butyl-2-hydroxybenzaldehyde (0.5 g) dissolved in ethanol (20 ml). Several drops of acetic acid were added. The solution was heated for 3 h. The solvent was evaporated and the product recrystallized from ethanol to yield orange prisms in 80% yield that were suitable for data collection.

### Refinement

Carbon-bound H-atoms were placed in calculated positions (C—H 0.95–0.98 Å) and were included in the refinement in the riding model approximation, with *U* iso(H) set to 1.2–1.5*U* eq(C).

The hydroxy H-atom was located in a difference Fourier map, and was refined with the O–H distance restrained to 0.84±0.01 Å; its temperature factor was refined.

In the final difference Fourier map, the largest peak was in the vicinity of an aromatic H-atom.

### Crystal data

**Table:**

C_24_H_31_NO_3_	*F*(000) = 824
*M**_r_* = 381.50	*D*_x_ = 1.197 Mg m^−^^3^
Monoclinic, *P*2_1_/*c*	Mo *K*α radiation, λ = 0.71073 Å
*a* = 18.4789 (18) Å	Cell parameters from 2280 reflections
*b* = 10.7194 (11) Å	θ = 2.2–23.8°
*c* = 10.7768 (10) Å	µ = 0.08 mm^−^^1^
β = 97.437 (2)°	*T* = 100 K
*V* = 2116.7 (4) Å^3^	Prism, orange
*Z* = 4	0.30 × 0.05 × 0.05 mm

### Data collection

**Table:**

Bruker SMART APEX diffractometer	3123 reflections with *I* > 2σ(*I*)
Radiation source: fine-focus sealed tube	*R*_int_ = 0.065
graphite	θ_max_ = 27.5°, θ_min_ = 2.2°
ω scans	*h* = −24→23
19941 measured reflections	*k* = −13→13
4855 independent reflections	*l* = −13→14

### Refinement

**Table:**

Refinement on *F*^2^	Primary atom site location: structure-invariant direct methods
Least-squares matrix: full	Secondary atom site location: difference Fourier map
*R*[*F*^2^ > 2σ(*F*^2^)] = 0.053	Hydrogen site location: inferred from neighbouring sites
*wR*(*F*^2^) = 0.142	H atoms treated by a mixture of independent and constrained refinement
*S* = 1.01	*w* = 1/[σ^2^(*F*_o_^2^) + (0.0558*P*)^2^ + 1.1473*P*] where *P* = (*F*_o_^2^ + 2*F*_c_^2^)/3
4855 reflections	(Δ/σ)_max_ = 0.001
257 parameters	Δρ_max_ = 0.64 e Å^−^^3^
1 restraint	Δρ_min_ = −0.25 e Å^−^^3^

### Fractional atomic coordinates and isotropic or equivalent isotropic displacement parameters (Å^2^)

**Table:**

	*x*	*y*	*z*	*U*_iso_*/*U*_eq_	
O1	0.78175 (8)	0.89233 (13)	0.62825 (14)	0.0252 (3)	
H1	0.7466 (13)	0.885 (3)	0.567 (2)	0.086 (12)*	
O2	0.42198 (8)	0.93692 (14)	0.03223 (14)	0.0291 (4)	
O3	0.43875 (7)	0.73467 (13)	−0.01081 (13)	0.0237 (3)	
N1	0.67954 (9)	0.79668 (16)	0.46645 (16)	0.0222 (4)	
C1	0.78970 (11)	0.77878 (18)	0.68363 (19)	0.0203 (4)	
C2	0.84179 (10)	0.76051 (18)	0.78972 (18)	0.0186 (4)	
C3	0.85050 (10)	0.63866 (18)	0.83515 (18)	0.0180 (4)	
H3	0.8862	0.6243	0.9054	0.022*	
C4	0.80999 (10)	0.53616 (18)	0.78384 (18)	0.0183 (4)	
C5	0.75592 (10)	0.56031 (19)	0.68469 (18)	0.0199 (4)	
H5	0.7253	0.4941	0.6509	0.024*	
C6	0.74560 (10)	0.67938 (19)	0.63368 (18)	0.0201 (4)	
C7	0.94204 (12)	0.9088 (2)	0.7566 (2)	0.0290 (5)	
H7A	0.9137	0.9359	0.6780	0.044*	
H7B	0.9735	0.8388	0.7402	0.044*	
H7C	0.9722	0.9782	0.7930	0.044*	
C8	0.88965 (11)	0.86740 (19)	0.84883 (19)	0.0219 (4)	
C9	0.84263 (13)	0.9784 (2)	0.8811 (2)	0.0295 (5)	
H9A	0.8124	1.0072	0.8051	0.044*	
H9B	0.8744	1.0464	0.9158	0.044*	
H9C	0.8112	0.9521	0.9428	0.044*	
C10	0.93590 (13)	0.8274 (2)	0.9709 (2)	0.0304 (5)	
H10A	0.9670	0.7569	0.9542	0.046*	
H10B	0.9037	0.8024	1.0320	0.046*	
H10C	0.9665	0.8974	1.0044	0.046*	
C11	0.82432 (11)	0.40232 (18)	0.82969 (18)	0.0197 (4)	
C12	0.75683 (11)	0.3511 (2)	0.8825 (2)	0.0254 (5)	
H12A	0.7465	0.4030	0.9529	0.038*	
H12B	0.7662	0.2652	0.9111	0.038*	
H12C	0.7148	0.3526	0.8169	0.038*	
C13	0.84080 (12)	0.3212 (2)	0.7192 (2)	0.0259 (5)	
H13A	0.8838	0.3540	0.6858	0.039*	
H13B	0.7988	0.3225	0.6535	0.039*	
H13C	0.8502	0.2352	0.7479	0.039*	
C14	0.88904 (11)	0.3930 (2)	0.9334 (2)	0.0247 (5)	
H14A	0.9330	0.4251	0.9025	0.037*	
H14B	0.8966	0.3056	0.9583	0.037*	
H14C	0.8789	0.4423	1.0057	0.037*	
C15	0.68922 (11)	0.69415 (19)	0.52586 (19)	0.0219 (4)	
H15	0.6589	0.6250	0.4995	0.026*	
C16	0.62487 (10)	0.8028 (2)	0.36008 (19)	0.0213 (4)	
C17	0.59190 (11)	0.9167 (2)	0.3314 (2)	0.0235 (5)	
H17	0.6062	0.9876	0.3815	0.028*	
C18	0.53831 (11)	0.9279 (2)	0.23032 (19)	0.0228 (5)	
H18	0.5157	1.0064	0.2116	0.027*	
C19	0.51707 (10)	0.82475 (19)	0.15535 (19)	0.0202 (4)	
C20	0.55214 (11)	0.7112 (2)	0.18075 (19)	0.0214 (4)	
H20	0.5391	0.6411	0.1286	0.026*	
C21	0.60615 (11)	0.7002 (2)	0.28224 (19)	0.0234 (5)	
H21	0.6305	0.6228	0.2990	0.028*	
C22	0.45526 (11)	0.84073 (19)	0.05346 (19)	0.0209 (4)	
C23	0.37799 (11)	0.7442 (2)	−0.1100 (2)	0.0265 (5)	
H23A	0.3908	0.8002	−0.1768	0.032*	
H23B	0.3347	0.7788	−0.0767	0.032*	
C24	0.36159 (12)	0.6163 (2)	−0.1613 (2)	0.0281 (5)	
H24A	0.3206	0.6204	−0.2286	0.042*	
H24B	0.3488	0.5616	−0.0945	0.042*	
H24C	0.4046	0.5830	−0.1944	0.042*	

### Atomic displacement parameters (Å^2^)

**Table:**

	*U*^11^	*U*^22^	*U*^33^	*U*^12^	*U*^13^	*U*^23^
O1	0.0265 (8)	0.0202 (8)	0.0271 (8)	−0.0029 (6)	−0.0030 (7)	0.0048 (6)
O2	0.0308 (8)	0.0244 (8)	0.0291 (8)	0.0063 (7)	−0.0075 (7)	0.0009 (7)
O3	0.0216 (7)	0.0233 (8)	0.0235 (8)	0.0036 (6)	−0.0069 (6)	−0.0009 (6)
N1	0.0198 (8)	0.0244 (9)	0.0225 (9)	0.0007 (7)	0.0031 (7)	0.0006 (8)
C1	0.0218 (10)	0.0195 (10)	0.0202 (10)	0.0052 (8)	0.0057 (8)	0.0059 (8)
C2	0.0173 (9)	0.0200 (10)	0.0187 (10)	0.0001 (8)	0.0031 (8)	0.0001 (8)
C3	0.0162 (9)	0.0229 (10)	0.0143 (9)	0.0016 (8)	−0.0008 (8)	0.0021 (8)
C4	0.0165 (9)	0.0206 (10)	0.0178 (10)	0.0008 (8)	0.0023 (8)	0.0009 (8)
C5	0.0173 (9)	0.0229 (11)	0.0191 (10)	−0.0008 (8)	0.0011 (8)	−0.0032 (8)
C6	0.0154 (9)	0.0273 (11)	0.0173 (10)	0.0025 (8)	0.0004 (8)	0.0000 (9)
C7	0.0294 (12)	0.0291 (12)	0.0286 (12)	−0.0085 (9)	0.0037 (10)	0.0017 (10)
C8	0.0252 (10)	0.0193 (10)	0.0204 (10)	−0.0026 (8)	0.0000 (8)	0.0017 (8)
C9	0.0393 (13)	0.0220 (11)	0.0273 (12)	−0.0012 (10)	0.0047 (10)	−0.0015 (9)
C10	0.0351 (12)	0.0253 (12)	0.0275 (12)	−0.0087 (10)	−0.0080 (10)	0.0024 (10)
C11	0.0201 (10)	0.0191 (10)	0.0192 (10)	0.0004 (8)	0.0002 (8)	−0.0015 (8)
C12	0.0262 (11)	0.0220 (11)	0.0280 (12)	−0.0025 (9)	0.0031 (9)	0.0009 (9)
C13	0.0309 (11)	0.0232 (11)	0.0232 (11)	0.0051 (9)	0.0023 (9)	−0.0025 (9)
C14	0.0269 (11)	0.0202 (11)	0.0254 (11)	0.0022 (9)	−0.0024 (9)	0.0009 (9)
C15	0.0212 (10)	0.0212 (11)	0.0235 (11)	−0.0011 (8)	0.0039 (8)	−0.0020 (9)
C16	0.0170 (9)	0.0283 (12)	0.0184 (10)	0.0001 (8)	0.0014 (8)	0.0009 (9)
C17	0.0216 (10)	0.0247 (11)	0.0238 (11)	−0.0014 (9)	0.0013 (9)	−0.0008 (9)
C18	0.0226 (10)	0.0227 (11)	0.0227 (11)	0.0021 (8)	0.0012 (9)	0.0015 (9)
C19	0.0187 (10)	0.0239 (11)	0.0174 (10)	0.0028 (8)	0.0004 (8)	0.0021 (8)
C20	0.0205 (10)	0.0228 (11)	0.0204 (10)	−0.0005 (8)	0.0016 (8)	0.0003 (9)
C21	0.0216 (10)	0.0241 (11)	0.0238 (11)	0.0060 (9)	0.0004 (8)	0.0039 (9)
C22	0.0194 (10)	0.0232 (11)	0.0198 (10)	0.0001 (8)	0.0012 (8)	0.0020 (9)
C23	0.0240 (11)	0.0295 (12)	0.0231 (11)	0.0018 (9)	−0.0082 (9)	0.0016 (9)
C24	0.0261 (11)	0.0306 (12)	0.0271 (12)	−0.0001 (10)	0.0012 (9)	−0.0006 (10)

### Geometric parameters (Å, °)

**Table:**

O1—C1	1.355 (2)	C11—C14	1.531 (3)
O1—H1	0.866 (10)	C11—C13	1.537 (3)
O2—C22	1.207 (2)	C11—C12	1.537 (3)
O3—C22	1.346 (2)	C12—H12A	0.9800
O3—C23	1.450 (2)	C12—H12B	0.9800
N1—C15	1.273 (3)	C12—H12C	0.9800
N1—C16	1.428 (3)	C13—H13A	0.9800
C1—C6	1.406 (3)	C13—H13B	0.9800
C1—C2	1.410 (3)	C13—H13C	0.9800
C2—C3	1.397 (3)	C14—H14A	0.9800
C2—C8	1.535 (3)	C14—H14B	0.9800
C3—C4	1.402 (3)	C14—H14C	0.9800
C3—H3	0.9500	C15—H15	0.9500
C4—C5	1.389 (3)	C16—C17	1.381 (3)
C4—C11	1.529 (3)	C16—C21	1.399 (3)
C5—C6	1.393 (3)	C17—C18	1.379 (3)
C5—H5	0.9500	C17—H17	0.9500
C6—C15	1.465 (3)	C18—C19	1.395 (3)
C7—C8	1.540 (3)	C18—H18	0.9500
C7—H7A	0.9800	C19—C20	1.389 (3)
C7—H7B	0.9800	C19—C22	1.488 (3)
C7—H7C	0.9800	C20—C21	1.387 (3)
C8—C10	1.534 (3)	C20—H20	0.9500
C8—C9	1.539 (3)	C21—H21	0.9500
C9—H9A	0.9800	C23—C24	1.494 (3)
C9—H9B	0.9800	C23—H23A	0.9900
C9—H9C	0.9800	C23—H23B	0.9900
C10—H10A	0.9800	C24—H24A	0.9800
C10—H10B	0.9800	C24—H24B	0.9800
C10—H10C	0.9800	C24—H24C	0.9800
			
C1—O1—H1	106 (2)	H12A—C12—H12B	109.5
C22—O3—C23	114.99 (16)	C11—C12—H12C	109.5
C15—N1—C16	118.83 (18)	H12A—C12—H12C	109.5
O1—C1—C6	119.20 (18)	H12B—C12—H12C	109.5
O1—C1—C2	120.37 (18)	C11—C13—H13A	109.5
C6—C1—C2	120.43 (18)	C11—C13—H13B	109.5
C3—C2—C1	116.73 (18)	H13A—C13—H13B	109.5
C3—C2—C8	121.24 (17)	C11—C13—H13C	109.5
C1—C2—C8	121.96 (18)	H13A—C13—H13C	109.5
C2—C3—C4	124.26 (18)	H13B—C13—H13C	109.5
C2—C3—H3	117.9	C11—C14—H14A	109.5
C4—C3—H3	117.9	C11—C14—H14B	109.5
C5—C4—C3	116.83 (18)	H14A—C14—H14B	109.5
C5—C4—C11	120.03 (17)	C11—C14—H14C	109.5
C3—C4—C11	123.12 (17)	H14A—C14—H14C	109.5
C4—C5—C6	121.49 (19)	H14B—C14—H14C	109.5
C4—C5—H5	119.3	N1—C15—C6	122.09 (19)
C6—C5—H5	119.3	N1—C15—H15	119.0
C5—C6—C1	120.03 (18)	C6—C15—H15	119.0
C5—C6—C15	117.32 (18)	C17—C16—C21	119.51 (19)
C1—C6—C15	122.63 (19)	C17—C16—N1	117.79 (19)
C8—C7—H7A	109.5	C21—C16—N1	122.64 (19)
C8—C7—H7B	109.5	C18—C17—C16	120.3 (2)
H7A—C7—H7B	109.5	C18—C17—H17	119.8
C8—C7—H7C	109.5	C16—C17—H17	119.8
H7A—C7—H7C	109.5	C17—C18—C19	120.5 (2)
H7B—C7—H7C	109.5	C17—C18—H18	119.7
C10—C8—C2	112.00 (17)	C19—C18—H18	119.7
C10—C8—C7	107.87 (18)	C20—C19—C18	119.40 (18)
C2—C8—C7	108.92 (17)	C20—C19—C22	122.69 (19)
C10—C8—C9	106.89 (17)	C18—C19—C22	117.87 (18)
C2—C8—C9	111.03 (17)	C21—C20—C19	120.0 (2)
C7—C8—C9	110.07 (18)	C21—C20—H20	120.0
C8—C9—H9A	109.5	C19—C20—H20	120.0
C8—C9—H9B	109.5	C20—C21—C16	120.18 (19)
H9A—C9—H9B	109.5	C20—C21—H21	119.9
C8—C9—H9C	109.5	C16—C21—H21	119.9
H9A—C9—H9C	109.5	O2—C22—O3	123.23 (18)
H9B—C9—H9C	109.5	O2—C22—C19	124.17 (19)
C8—C10—H10A	109.5	O3—C22—C19	112.57 (17)
C8—C10—H10B	109.5	O3—C23—C24	107.99 (17)
H10A—C10—H10B	109.5	O3—C23—H23A	110.1
C8—C10—H10C	109.5	C24—C23—H23A	110.1
H10A—C10—H10C	109.5	O3—C23—H23B	110.1
H10B—C10—H10C	109.5	C24—C23—H23B	110.1
C4—C11—C14	112.55 (16)	H23A—C23—H23B	108.4
C4—C11—C13	108.92 (16)	C23—C24—H24A	109.5
C14—C11—C13	108.30 (16)	C23—C24—H24B	109.5
C4—C11—C12	109.85 (16)	H24A—C24—H24B	109.5
C14—C11—C12	107.53 (17)	C23—C24—H24C	109.5
C13—C11—C12	109.64 (17)	H24A—C24—H24C	109.5
C11—C12—H12A	109.5	H24B—C24—H24C	109.5
C11—C12—H12B	109.5		
			
O1—C1—C2—C3	−175.11 (17)	C3—C4—C11—C13	−122.2 (2)
C6—C1—C2—C3	4.6 (3)	C5—C4—C11—C12	−64.1 (2)
O1—C1—C2—C8	1.8 (3)	C3—C4—C11—C12	117.6 (2)
C6—C1—C2—C8	−178.52 (18)	C16—N1—C15—C6	178.21 (17)
C1—C2—C3—C4	−1.5 (3)	C5—C6—C15—N1	−174.29 (19)
C8—C2—C3—C4	−178.36 (18)	C1—C6—C15—N1	4.0 (3)
C2—C3—C4—C5	−2.7 (3)	C15—N1—C16—C17	151.3 (2)
C2—C3—C4—C11	175.58 (19)	C15—N1—C16—C21	−31.4 (3)
C3—C4—C5—C6	3.9 (3)	C21—C16—C17—C18	3.2 (3)
C11—C4—C5—C6	−174.44 (18)	N1—C16—C17—C18	−179.46 (18)
C4—C5—C6—C1	−0.9 (3)	C16—C17—C18—C19	−0.4 (3)
C4—C5—C6—C15	177.43 (18)	C17—C18—C19—C20	−2.2 (3)
O1—C1—C6—C5	176.18 (18)	C17—C18—C19—C22	175.61 (19)
C2—C1—C6—C5	−3.5 (3)	C18—C19—C20—C21	2.0 (3)
O1—C1—C6—C15	−2.1 (3)	C22—C19—C20—C21	−175.68 (18)
C2—C1—C6—C15	178.18 (18)	C19—C20—C21—C16	0.8 (3)
C3—C2—C8—C10	−9.4 (3)	C17—C16—C21—C20	−3.4 (3)
C1—C2—C8—C10	173.86 (19)	N1—C16—C21—C20	179.43 (18)
C3—C2—C8—C7	109.8 (2)	C23—O3—C22—O2	0.8 (3)
C1—C2—C8—C7	−66.9 (2)	C23—O3—C22—C19	178.95 (17)
C3—C2—C8—C9	−128.8 (2)	C20—C19—C22—O2	177.1 (2)
C1—C2—C8—C9	54.5 (2)	C18—C19—C22—O2	−0.6 (3)
C5—C4—C11—C14	176.14 (18)	C20—C19—C22—O3	−1.0 (3)
C3—C4—C11—C14	−2.1 (3)	C18—C19—C22—O3	−178.74 (18)
C5—C4—C11—C13	56.0 (2)	C22—O3—C23—C24	−173.52 (17)

### Hydrogen-bond geometry (Å, °)

**Table:**

*D*—H···*A*	*D*—H	H···*A*	*D*···*A*	*D*—H···*A*
O1—H1···N1	0.87 (1)	1.80 (2)	2.609 (2)	154 (3)
